# Virtual Reality-Integrated Immersion-Based Teaching to English Language Learning Outcome

**DOI:** 10.3389/fpsyg.2021.767363

**Published:** 2022-02-08

**Authors:** Yu Xie, Yang Liu, Fengrui Zhang, Ping Zhou

**Affiliations:** ^1^School of Foreign Languages, Jiangnan University, Wuxi, China; ^2^Institute of Education, University College London, London, United Kingdom; ^3^Trinity College, University of Cambridge, Cambridge, United Kingdom; ^4^School of Foreign Studies, Hunan University of Humanities, Science and Technology, Loudi, China

**Keywords:** virtual reality, immersive language teaching, English language, learning outcome, language ability

## Abstract

Globalization and informatization are reshaping human life and social behaviors. The purpose is to explore the worldwide strategies to cultivate international talents with a global vision. As a global language with the largest population, English, and especially its learning effect, have always been the major concerns of scholars and educators. This work innovatively studies the combination of immersion-based English teaching with virtual reality (VR) technology. Then, based on the experimental design mode, 106 students from a Chinese school were selected for a quasi-experimental study for 16 weeks (3 h a week, and 48 h in total). The collected data were analyzed by computer statistical software, and hypotheses are verified. The results showed that there is a significantly positive correlation between VR and immersion-based language teaching (0.851, *p* < 0.01). There is a significantly positive correlation between immersion-based language teaching and academic achievement (0.824, *p* < 0.01), and VR is positively correlated with learning outcome (LO) (0.836, *p* < 0.01). Compared with other state-of-art research methods, this work modifies the students’ oral test through the analysis and comparison with the system database, and the students’ learning effect is greatly improved. Finally, some suggestions are put forward according to the research results to provide an experimental reference for English teachers and future linguistics teaching.

## Introduction

The era of globalization and informatization is witnessing a profound revolution in human civilization and the relationship of humans, nature, and various social groups. Under such backgrounds, education of international talents with global vision is the key to successful educational systems in every nation. Language proficiency level (LPL) is not just the symbol of personal identity and status, but also has become an important educational issue. Foreign language teaching (FLT) is promoted by favorable government policies and a successful learning and teaching model (LTM). According to the American foreign language learning (FLL) standards, FLL covers five objectives, such as communication, culture, connection, comparison, and community, known as 5C. FLT aims to make learners use foreign language (FL) for communication in the fields of listening, speaking, reading, and writing to express personal thoughts, emotions, and opinions; experience diverse cultures; and appreciate the history and culture of the target language (TL). For this reason, FL should not be regarded as a subject for repeatedly reciting new words and practicing grammar. Instead, FL can be used for reinforcing the learning of other subjects and comparing the characteristics of native language (NL) and TL, as well as distinct language culture. The learning should not be restricted to school environments but extended to the international society for cultivating lifelong learners.

The spirit of immersion-based teaching corresponds to the American 5C FLL standards and presents a global aspect and cross-culture FLL methods. The US government actively promotes immersion-based Chinese as a second language (CSL) teaching in 2001. According to native language acquisition, the course integrates language and cognition, allowing students to learn Chinese in the near authentic situation. Moreover, as artificial intelligence (AI) develops, AI is more widely applied in education, for example, in the selection and application of teaching media, the analysis of learners’ learning characteristics, the design of learners’ autonomous learning mode, and the improvement of teaching resources, AI can be used to complete the corresponding services to realize the intelligent development of education and personalized teaching of students. The traditional speech recognition (SR) technology can only recognize an individual’s voice from a close range and also has strict requirements on pronunciations and the ambient environments. The continuous maturation of AI helps to solve many problems in SR technology by enabling many fields to use audio files to output corresponding Chinese characters to complete more work. Under the global CSL learning fever, courses of the CSL, as well as the English as a second language (ESL), have been gradually established and matured. Canada and the US are the pioneers of immersion-based CSL teaching. In consideration of the external conditions of the global economy, China has become the top world economic power in the twenty-first century. Therefore, CSL teaching and learning have become an international trend, and more and more foreigners choose to settle down or start businesses in China. The number of foreigners in China is closely related to the CSL learning market. The increased learners’ needs and diverse backgrounds also indirectly help update the quality and pattern of CSL teaching. For example, Halabi (2020) strengthened engineering education and teaching with immersion-based virtual reality (VR) technology. The results showed that VR reduced the course development time and cost, while enhancing students’ enthusiasm and creativity. Compared with traditional methods, the VR method improved students’ communication and problem-solving abilities. McCleery et al. (2020) taught autistic adolescents by immersion-based VR intervention. The research results indicated that immersion-based VR might provide an intervention mechanism for the social skills of the autistics, but it was unclear whether the anxiety concerns or sensory symptoms would limit the feasibility of the intervention. Carbonell-Carrera et al. (2021) studied the user VR experience in the immersion-based three-dimensional (3D) visualization environment. The research found that in the nine subcategories analyzed (sense of presence, sense of participation, sense of immersion, flow, usability, emotion, judgment, experience consequences, and technology adoption), the perception of the 3D environment in the visualization process was very high.

Therefore, the combination of immersion-based FLT and VR can be used to study the effect of English learning. Moreover, the application of VR technology in AI to students’ oral training and homework correction provides a more convenient tool for English Majors’ oral practice. Then, based on the experimental design model, 106 English major students from a Chinese school were selected for a quasi-experimental study for 16 weeks (3 h a week, and a total of 48 h). Innovatively, this work presents the combination of immersion-based English teaching with VR technology. Then, the effectiveness of the proposal is verified by testing various hypotheses. The first section introduces the background of second language learning and the characteristics of immersion-based learning. The second section, Literature review, further expounds on the research status of VR and immersion-based FLT. Then, the third section carries out sample measurement and index evaluation; and after the reliability and validity test, the experimental simulation design is completed. The fourth section analyzes the empirical results and implements the linear structure model. Finally, the fifth chapter summarizes the experimental conclusions. The relevant suggestions of immersive FLT have practical reference value for English teachers and scholars to improve the effect of ESL teaching. Shadiev and Sun (2020) applied the combination of speech-to-text recognition technology in English as a foreign language lecture to promote students’ understanding of the lecture content.

## Literature Review

### Virtual Reality

Lin (2017) explained that VR, also called artificial environment, is generating a 3D virtual world with computer simulation and providing users with a sensory stimulation of sight, hearing, and touch which allows users to timely observe affairs and objects in the 3D space without restrictions. Richardson et al. (2017) pointed out that VR was the feeling of presence in a virtual environment, namely, the artificial environment. VR simulated a 3D scene for real-time response through human–computer interaction (HCI) so that people could feel the presence. Archer and Finger (2018) simply explained VR as creating a virtual world with computer graphics, allowing users to have authentic feelings. Herrera et al. (2018) mentioned that VR constructed the environment through computer equipment and integrated authentic or virtual pictures into simulated situations to present the authentic effect. The environment presented highly authentic interaction, allowing users to view various computer images or videos and operate or interact with the objects through HCI. Users could freely move in the space to appear senses of integration and participation to further feel the presence.

According to Wu and Chen (2018), VR covers 3 dimensions in this study.

1. Imagination: It allows people to connect with their experiences, after receiving the sensory stimulation, to generate the illusion between true and false.
2. Immersion: It allows people to experience the entire VR with senses to integrate into the virtual world and feel the presence.
3. Interactivity: It allows people to interact with virtual objects as in the real world. Apparently, VR integrates people into the 3D virtual environment simulated by computers. The real-time simulation and interaction through HCI offer users the feeling of presence.

There have been various works on the research of VR. For example, Lv et al. (2020) argued that VR technology could be used to design industrial security solutions. The constructed algorithm performed well in the attack response to the industrial control system in the VR simulation environment, which provided practical significance for the application of VR technology. Chang et al. (2020) proposed various indexes to accurately measure the disease level by reviewing the measurement of VR disease symptoms and causes. Emmelkamp and Meyerbröker (2021) used VR technology to diagnose and treat mental health diseases, and the research showed that the application of VR in phobia could be extended to the diagnosis and treatment of other mental health disorders.

### Immersion-Based Foreign Language Teaching

Kao et al. (2016) indicated that immersion-based teaching promoted the idea of a language swimming pool, allowing students to cultivate their language sense in the environment with the pure TL. Students, either in class/after class or on/off-campus, would completely immerse themselves in the TL ocean, constantly receive the stimulation of distinct TL information, absorb TL knowledge, repeatedly practice TL skills, and continuously use TL for learning and living to eventually develop TL thinking pattern and expression ability naturally. Slater (2018) stated that interaction and communication were the major teaching forms, the classroom climate was relaxing and could fully induce students’ motivation for positive participation, and students could cultivate the habit of thinking with TL in the real immersive environment, break through their psychological barriers, and pick up some language skills. Bachen et al. (2016) explained the so-called immersion-based teaching that students, for about all or half of their time in schools, immersed in the English language environment, teachers taught language knowledge using English, and the English language was used for teaching non-language courses as well. In other words, the English language was the learning content as well as the learning tool. La Corte et al. (2019) regarded immersion-based teaching as a teaching method for learning English, which mainly used TL for general teaching activities. TL was the medium of teaching, rather than the major objective and object in classroom teaching. Hortensius et al. (2018) pointed out the core concept of immersion-based language teaching as using the function and objective of language for naturally learning language, as the initial function, and objective of language were for message communication. Immersion-based language teaching is expected to replace the NL with TL in thinking or language application environments; when learners were immersed in the language application environment for a long period, their sense of TL could be naturally cultivated through interaction with people using TL.

According to Lin et al. (2018), FLL should be content-based. Full exposure to the meaningful language input situation is the key to acquiring language. Language learning and teaching contain three dimensions.

1. General characteristics: It means to explore the association between pronunciation and word, regional and general accent, grammar, and vocabulary.
2. Teaching strategy: It means to develop active language skills through the application of individual prior knowledge to language and promote children’s LPL by discussing and analyzing distinct text contents.
3. Actual application: It allows children to acquire grammar under the structure of sense of language. The developed grammar does not simply focus on the general language but expects to deepen students’ sense of language and awareness of language in teaching activities or learning tasks.

To sum up, there are many studies on immersion-based language teaching. For example, Altun and Lee (2020) used meta-analysis to carry out immersion-based English language teaching. The research results showed that immersion-based learning technology had a positive impact on English language learning. Wang et al. (2021) employed immersive experience technology to improve the effect of undergraduate Chinese multilevel learning. The results suggested that students liked to explore experiential learning opportunities of English culture and language outside the classroom. Hayes et al. (2021) studied the immersion degree of interactive VL language learning and mapped the immersion level to the learning objectives related to language learning through replicable simulation and social interaction. Combined with the above research, the use of immersion-based teaching can effectively improve the effect of English teaching. Kim et al. (2019)’s research shows that robot programs allow direct interaction between human and computer.

### Artificial Intelligence and Speech Recognition

Cerezo et al. (2019) described a mobile phone application, which helped Spanish children practice the pronunciation of basic English vocabulary, and the robot served as a virtual teacher and interacted with children by dividing them into the experimental group and the control group. According to the performance evaluation before and after the test, satisfaction survey, and children’s emotional analysis, the application significantly affected children’s English learning. Bin and Mandal (2019) pointed out that the application of computer technology (CT) and AI technology in the field of education would become a trend, even in the automatic scoring of English composition. Specifically, based on the integration of information technology and curriculum, the application of AI in middle school English teaching was explored by combining curriculum theory, literature analysis, and field investigation. Moreover, the implementation scheme of English assisted teaching system based on AI technology was proposed. The results also implied that AI technology could make the teaching system more humanized and further improve the quality and effect of English teaching. Park et al. (2020) studied the improved automatic SR system, and the recognition accuracy could be improved by 4% on noisy experimental test data sets. Ye et al. (2021) studied the dual-channel hybrid-level framework using SR; the system reduced the relative word error rate by 8–10%, showing that the proposed system might maintain the advantages of the hybrid system. In conclusion, the application of AI in SR has practical reference value for immersion-based English learning and teaching.

### Learning Outcome

Hong et al. (2016) mentioned that teachers could understand the practice effect of teaching activity from students’ evaluation results and further inspect the teaching excellence for evaluating teaching. Teachers could understand students’ responses to different teaching methods by observing their learning responses and understanding their performance in the class or learning process. Isabelli-García and Lacorte (2016) regarded learning outcome (LO) as the practice of various types of evaluation or tests on learners for a period of time after learning activity to understand their LO on the learned content. Lull and Bushman (2016) pointed out LO as an indicator to evaluate students’ absorption of course content; and, teachers’ teaching effectiveness could be judged based on students’ test performance. Chrysikou and Thompson (2016) regarded LO as students’ change before and after receiving education. The real change was by deducting “entry behavior” before education from “terminal behavior” after education, which was the students’ “direct” LO. On the contrary, students’ “indirect” LO was the effect appearing a period after students had been educated. van Loon et al. (2018) stated that students’ LO was inspected through examinations to check whether students mastered the knowledge and skills preset in the teaching objectives and learned teaching activities or materials. The higher test performance revealed students’ better LO.

According to Tsai et al. (2018), the following factors in students’ LO are proposed in this study.

1. Teaching factors: They include peer relationships, student–teacher interaction, classroom equipment, learning environment, family background, and cultural value of community culture.
2. Environment factors: They include teaching approaches, teaching time, curriculum design, material content, and teachers’ organization and interpretation ability.

Meanwhile, concerning other factors affecting students’ academic performance, Liu et al. (2020) conducted a meta-analysis on the factors affecting students’ academic performance. The results corroborate that there was a medium relationship between socio-economic status and academic performance. Sadeqi et al. (2021) studied the impact of curriculum teaching on students’ learning effects. The research results revealed that teaching practical knowledge by comprehensive methods was of great help to students’ clinical experimental application.

### Research Hypotheses

Aiming at abstract concept learning and specific operation learning, Wenga et al. (2018) conducted the clinical teaching for experimental comparison. The experiment proved that learning anxiety and frustration could be easily alleviated when pictures were applied to operational abstract concept learning. Besides, the logical thinking in programmed learning was not simple, and actual active operation learning could acquire sensory experience of seeing and touch and appear better message processing than learners with abstract ideas. DeSmet et al. (2016) applied the characteristics of virtual space and further used innovative immersive interaction media to prove that virtual operation through users’ intuitive allowed users to not only acquire information from picture-based abstract concept learning but also integrate the interactivity of immersion-based VR. Wu and Chen (2018) discovered that the application of VR could make up for the inadequacy of other teaching methods through the interactive virtual learning environment; it could enhance learners’ enthusiasm and provide interactive teaching, as well as apply the space of virtual environment to promote immersion-based teaching on the spatial structure. Chen et al. (2021) used interactive spherical video to study the writing of VR Chinese composition teaching. The results proved that it could improve students’ writing ability. Accordingly, the following hypothesis is inferred.

H1: VR presents positive relations with immersion-based language teaching.

Shin (2018) indicated that immersion-based teaching allowed learners to learn the course content through a TL environment, so it could eventually help learners become bilingual learners; for this reason, the use of TL between teachers and students was particularly emphasized. Madsen (2016) analyzed the effect of immersion-based listening and reading learning period on the learning effect of elementary pupils’ ESL learning. It was discovered that longer immersive courses for either English listening or reading appeared to produce a better performance on foreign language acquisition. Ferchaud and Sanders (2018) proposed that immersion-based English teaching could enhance elementary pupils’ learning attitudes and English test performance. It was, therefore, considered that immersion-based learning could assist in enhancing English learning effectiveness. Lin et al. (2018) discovered that pupils receiving entirely immersion-based English learning showed higher learning confidence, keener learning interests, and also stronger learning belief than those who did not. Chan et al. (2021) applied immersive VR to distance architectural history teaching. The research results signified that immersive visualization ability could be used as an essential distance teaching tool in educational institutions to improve students’ learning effects. In this case, the following hypothesis is inferred.

H2: Immersion-based language teaching shows positive relations with LO.

Zych et al. (2019) mentioned that VR stressed being on the scene, and the sensory stimulation of the taught was far more than picture and text message delivery on print media to deepen the topic memory and comprehension; it, therefore, could effectively promote LO. Pavone et al. (2016) indicated that a lifelike simulation situation could induce students’ learning motivation, implement the learning initiative, and control the interaction with the environment to achieve the skill learning and effectively enhance LO. Gorisse et al. (2017) stated that computer simulation technology offered learners with proper control; learners could control the learning sequence, content, and schedule and actively operate, explore, and recombine knowledge. It conformed to the spirit of constructive learning to promote LO. Tsai et al. (2018) indicated that a well-designed skill simulation system could simulate authentic operation situations, allowing learners to clearly learn important concepts through self-control, interaction, simulation, and observation, thereby effectively enhancing the LO. Han et al. (2021) conducted the inspection and research of nervous system teaching tools based on VR. The research results implied that VR technology could effectively improve students’ learning satisfaction. The following hypothesis is therefore inferred in this study.

H3: VR reveals positive relations with LO.

Knox (2020) pointed out that AI played a great role in educational institutions. New Oriental Group, tomorrow advancing life (TAL), and squirrel AI institutions have made good achievements in the use of AI. They also stated that educational institutions were influential participants in national and regional AI development strategies. Cope et al. (2020) pointed out that AI would never take over the role of teachers, but it could be used to improve the potential of education in a certain range and make education more humanized. Lin and Wang (2021) used VR technology in English language learning. The research found that VR technology could be effectively integrated into English classrooms to promote students’ creative self-efficacy and intrinsic motivation. Therefore, the following assumption is deduced:

H4: AI technology can improve students’ oral English.

## Sample and Measurement Indicators

### Research Sample and Object

With the experimental design model, a total of 106 English-major students in a Chinese school were selected as the research objects for the 16-week (3 h per week, with a total of 48 h) quasi-experimental teaching research. The retrieved data are analyzed with computer statistics software to test various hypotheses. Combined with the research results of Schieber et al. (2018), the sample form of the designed questionnaire survey (QS) is manifested in Table 1. After the students’ study, the QS is distributed for statistics, and the learning effect is analyzed after unified recovery and processing.

**TABLE 1:** A QS on the effectiveness of VR technology in English language learning.

Do you know or have contact with VR technology?		

A. I don’t know.	B. I know something	C. I know very well.
Does the lack of learning context affect English language learning?		
A. Have no effect.	B. Have a certain impact.	C. Have a great impact.
What is your English LPL?		
A. It is difficult to communicate with British and American people.	B. It is good for simple communication with British and American people.	C. It can be used to communicate smoothly with British and American people.
Have you improved your oral English proficiency after using oral correction software for a period of time?		
A. Have not improved at all.	B. Have slightly improved.	C. Have greatly improved.

### Reliability and Validity Test

Validity refers to a measurement tool to truly measure what a researcher intends to measure. Generally speaking, validity is divided into content validity, criterion-related validity, and construct validity. The QS design in this work has referred to domestic and international researchers’ research items that the QS shows certain content validity. Dimensions of VR, immersion-based language teaching, and LO in this work are tested through the overall structural causality with linear structural relation (LISREL). The data input is based on the correlation coefficient matrix of the above observatory variables. The analysis results with the LISREL model reveal that the overall model fit reaches the reasonable range that it presents favorable convergent validity and predictive validity. Item-to-total correlation coefficients are used for testing the construct validity of the QS content, i.e., reliability analysis. The calculated item-to-total correlation coefficients are used for judging the questionnaire content. The item-to-total correlation coefficients of dimensions in the designed QS are higher than 0.7, revealing a certain degree of the construct validity of the dimensions.

Reliability and validity analyses are conducted to further understand the reliability and validity of the QS. The higher Cronbach’s α shows the better reliability. The formal QS is developed according to the standard, and the measured Cronbach’s α appears in 0.75–0.90, apparently conforming to the reliability range.

### Oral Test Correction

After the content data of the students’ oral test are collected, the data are transformed to extract the characteristic value of the speech content conveniently. Through identifying the features of vocabulary, sentence, and text structure, and then correcting the final learning results, the effectiveness of immersion-based language teaching is tested.

The high-frequency words expressed in oral English are input into the intelligent system. With a full score of 10 points, the contents are scored by the system according to their correlation degree. The scores are recorded in the system for the following score collection.

### Experimental Environment

With VR equipment, the students can have immersive learning independently in a classroom, in which there are several small spaces for the experiment. Therefore, each student can log in to the account before using it and perform voice interaction in the independent space without being disturbed by the external environment. After the students log in, the teacher can check the students’ learning and training at any time by logging into the background system to assist the students in completing the follow-up study.

**FIGURE 1:**
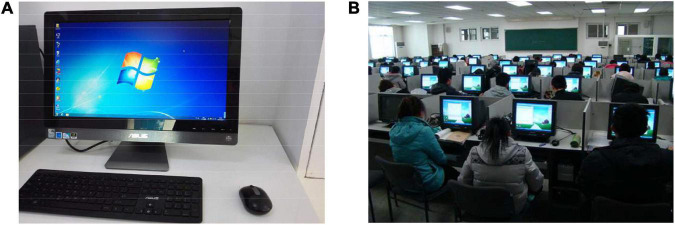
Specific environmental conditions (**A:** A single computer used in the student simulation experiment; **B:** Scene of the student simulation experiment).

The students can also learn through the mobile APP. The contents of the mobile APP can be synchronized with those in the experimental classroom system so that the students can learn relevant content at any time. During classes, VR equipment is used for immersion-based learning to consolidate what the students have learned. The specific environmental conditions are manifested in Figure 1.

## Empirical Results Analysis

### LISREL Model Evaluation Indicator

In the LISREL model, the factor analysis is combined with the path analysis in traditional statistics, and simultaneous equations are added in econometrics for simultaneously calculating multiple factors and multiple causal paths. The evaluation of model fit can be evaluated from the preliminary fit criteria, overall model fit, and fit of the model internal structure.

The data are organized as shown in Table 2, and the preliminary fit criteria, internal fit, and overall fit of the model are explained as follows.

**TABLE 2:** Overall LISREL model analysis result.

Evaluation item	Parameter/evaluation standard		Result		t
Preliminary fit	VR	Imagination	0.741		11.29**
		Immersion	0.723		10.17**
		Interactivity	0.758		12.63**
	Immersion-based language teaching	General characteristics	0.706		8.66**
		Teaching strategy	0.735		10.53**
		Actual application	0.712		9.42**
	LO	Teaching factors	0.766		13.28**
		Environment factors	0.783		15.24**
Internal fit	VR → Immersion-based language teaching		0.832		25.37**
	Immersion-based language teaching → LO		0.873		33.71**
	VR → LO		0.856		29.16**
Overall fit	X2/Df			1.626	
	GFI			0.974	
	AGFI			0.932	
	RMR			0.004	

* stands for p < 0.05, ** for p < 0.01, and *** for p < 0.001.

Table 2 shows that three dimensions (imagination, immersion, and interactivity) can significantly explain VR (*t* > 1.96, *p* < 0.05). The three dimensions (general characteristics, teaching strategy, actual application) can remarkably explain immersion-based language teaching (*t* > 1.96, *p* < 0.05); and two dimensions (teaching factors and environment factors) of can notably explain the LO (*t* > 1.96, *p* < 0.05). Apparently, the overall model reveals favorable preliminary fit criteria.

Regarding internal fit, VR presents positive and significant correlations with immersion-based language teaching (0.851, *p* < 0.01); immersion-based language teaching shows positive and remarkable correlations with LO (0.824, *p* < 0.01); VR appears positive and notable correlations with LO (0.836, *p* < 0.01); thus, H1, H2, and H3 are supported.

Regarding the overall model fit, the overall model fit standards χ^2^/Df 1.626 indicates it is smaller than the standard value 3; RMR 0.004 reveals the proper χ^2^/DF and RMR results. Furthermore, the chi-square value is sensitive to sample size, and so it is not suitable for directly judging the fit. However, the overall model fit standards GFI 0.974 and AGFI 0.932 are above 0.9 (the closer GFI and AGFI are to 1, the better model fit is); thus, this model presents better fit indices. The results of hygiensis test are shown in Table 3.

**TABLE 3:** Hypothesis test.

Research hypothesis	Correlation	Empirical result	*P*	Result
H1	+	0.832	*p* < 0.01	Supported
H2	+	0.873	*p* < 0.01	Supported
H3	+	0.856	*p* < 0.01	Supported

### Students’ Oral Test Correction

In the process of correction, the SR technology is used to extract the relevant content in the audio content of the oral test, including the characteristics of vocabulary, sentence, and text structure. Through the analysis and comparison with the system database, the students’ oral test is corrected, and the corresponding results and suggestions are given.

**FIGURE 2:**
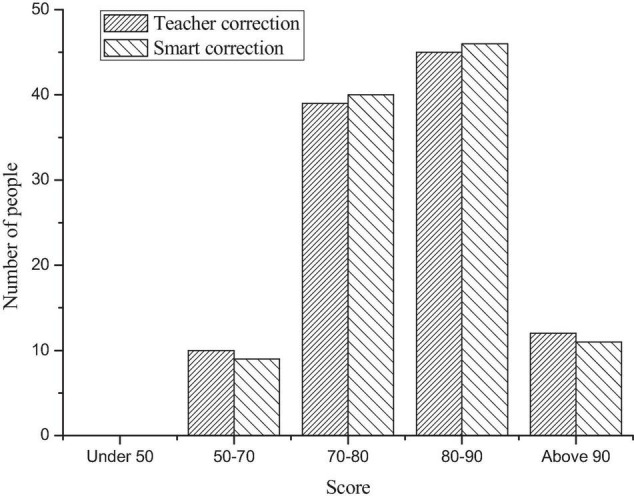
Score distribution.

Among the 106 oral test audios submitted, the corresponding oral test scores are shown in Figure 2.

Figure 2 shows that most people scored 80–90, followed by 70–80. Moreover, there is a small score gap between the teacher’s correction and AI grading, which can prove that it is feasible and effective to use AI for correction.

Meanwhile, AI is used to make a correction, and the system also gives suggestions for revision, such as word pronunciation, sentence structure, and logic of the text. This requires students to focus on the pronunciation of words and the grammatical relationship of sentences when practicing oral English, thus further conveying the correct pronunciation to the other side while improving the expression ability and avoiding misunderstanding in the communication.

Simultaneously, with an extensive voice database, the AI system provides students with reading examples and rereading choices. Various functions, such as reading and accent correction, can also be realized. The research results indicate that a deep understanding of their deficiencies can be provided for the students of different English proficiency by the AI system within a short time. Meanwhile, after the students complete the corresponding learning tasks within a specified time, their learning results are recorded automatically and analyzed by the system. Then more targeted exams are arranged for them. Students can apply their knowledge through frequent tests of what they have learned. In this way, teaching and learning are completed. Besides, the system also provides students with analyses of focuses and difficulty. Through suggested answers to and remarks on important knowledge, the students can finish the training. Besides, homework search and problem analysis can be completed in the AI system’s homework management module so that the students can see their disadvantages clearly.

## Discussion

Experimental results imply that there is a positive and significant correlation between VR and immersion-based language teaching. Additionally, the use of SR technology for students’ oral tests and correction and the immersion-based VR teaching method can enable students to improve their oral English skills. AI technology is applied to oral English learning, training, and testing. By analyzing the oral test content and test results, students can apply their knowledge to life. Continuous learning and testing help to systematically and clearly understand students’ oral English skills.

Immersion-based English teaching can have a positive impact on ESL learning. For example, Altun and Lee (2020) researched immersion-based learning technology in ESL teaching. The research revealed that immersion-based learning technology could have a positive impact on ESL teaching. Abdullah and Mohamad (2020) researched the implementation of a highly immersive curriculum on primary school students’ language ability and argued that it encouraged students to learn English in an effective English environment. Meanwhile, the research found that students welcomed experiential learning opportunities to explore foreign cultures and languages outside the classroom. Through the formulation of a language learning plan, students’ oral ability had been effectively developed. Therefore, AI would be more widely used in oral English teaching in the future.

## Conclusion

The research results show that the application of immersion-based VR English teaching can help students continuously improve their English LPL in listening, speaking, reading, and writing. This is because, during immersion-based teaching, teachers use English as much as possible to confer relevant knowledge, while students also use English to communicate and think the way English native speakers do to better understand the teaching content. In other words, when students learn English under meaningful situations, the teaching effect could be significantly enhanced. The application of VR to immersion-based English language teaching presents such an excellent effect on the quantity of students’ learning that they can learn more within the same learning time. It is also indicated that after VR is used for the preview of immersion-based English teaching, students can better understand the teaching content. In comparison with teachers preceding one-way teaching, actual operation and communication interaction can be directly conducted by reviewing the content before classes. Therefore, the proposed method can largely enhance students’ learning speed. There will also be a good performance by using AI-based SR technology to identify the oral test content, and the feedback can help students correct the wrong pronunciation in language expression and improve their oral ability.

## Implications for Academic Contributions and Practices

Based on the research results and findings, the following practical suggestions are proposed in this study.

1. Professional communities could be organized to help English language teachers improve their professional competency with the assistance of groups so that teachers can practice different teaching strategies with proper attitudes and competency. Moreover, mutual learning and encouragement in groups can improve teaching attitudes and transform teaching beliefs, so it is favorable for enhancing the passion for more active teaching.
2. The application of immersion-based teaching strategies by Chinese language teachers is helpful to enhance students’ Chinese language proficiency. For learners with different levels, the selection of proper immersion-based teaching strategies could better conform to individual learning needs and confer more cultural knowledge.
3. The application of VR to immersion-based language teaching for preview would benefit students’ LO. Ease of use is emphasized in technology, and the future design would become simpler. Teachers with good computer skills can devise well-matched digital materials with the teaching content to save resources and constantly offer new ideas to enrich learning.
4. The use of AI technology can help students improve their oral English proficiency. Moreover, more AI technologies can be employed to further improve the teaching methods, such as HCI and deep learning (DL). In this case, students can use advanced technology to complete the course content in a relaxed environment. Meantime, these technologies can also make students more active to learn and practice, thus truly realizing students’ autonomous learning, and positively affecting the development of the education industry.

## Data Availability Statement

The raw data supporting the conclusions of this article will be made available by the authors, without undue reservation.

## Ethics Statement

The studies involving human participants were reviewed and approved by the Jiangnan University Ethics Committee. The patients/participants provided their written informed consent to participate in this study. Written informed consent was obtained from the individual(s) for the publication of any potentially identifiable images or data included in this article.

## Author Contributions

All authors listed have made a substantial, direct, and intellectual contribution to the work, and approved it for publication.

## Conflict of Interest

The authors declare that the research was conducted in the absence of any commercial or financial relationships that could be construed as a potential conflict of interest.

## Publisher’s Note

All claims expressed in this article are solely those of the authors and do not necessarily represent those of their affiliated organizations, or those of the publisher, the editors and the reviewers. Any product that may be evaluated in this article, or claim that may be made by its manufacturer, is not guaranteed or endorsed by the publisher.
